# Safety and image quality at 7T MRI for deep brain stimulation systems: Ex vivo study with lead-only and full-systems

**DOI:** 10.1371/journal.pone.0257077

**Published:** 2021-09-07

**Authors:** Bhumi Bhusal, Jason Stockmann, Bastien Guerin, Azma Mareyam, John Kirsch, Lawrence L. Wald, Mark J. Nolt, Joshua Rosenow, Roberto Lopez-Rosado, Behzad Elahi, Laleh Golestanirad

**Affiliations:** 1 Department of Radiology, Northwestern University, Chicago, IL, United States of America; 2 Department of Radiology, Harvard Medical School, Boston, MA, United States of America; 3 Athinoula A. Martinos Center for Biomedical Imaging, Massachusetts General Hospital, Boston, MA, United States of America; 4 Department of Neurosurgery, Northwestern University, Chicago, IL, United States of America; 5 Department of Physical Therapy and Human Movement Sciences, Northwestern University, Chicago, IL, United States of America; 6 Department of Biomedical Engineering, Northwestern University, Evanston, IL, United States of America; National Institutes of Health, UNITED STATES

## Abstract

Ultra-high field MRI at 7 T can produce much better visualization of sub-cortical structures compared to lower field, which can greatly help target verification as well as overall treatment monitoring for patients with deep brain stimulation (DBS) implants. However, use of 7 T MRI for such patients is currently contra-indicated by guidelines from the device manufacturers due to the safety issues. The aim of this study was to provide an assessment of safety and image quality of ultra-high field magnetic resonance imaging at 7 T in patients with deep brain stimulation implants. We performed experiments with both lead-only and complete DBS systems implanted in anthropomorphic phantoms. RF heating was measured for 43 unique patient-derived device configurations. Magnetic force measurements were performed according to ASTM F2052 test method, and device integrity was assessed before and after experiments. Finally, we assessed electrode artifact in a cadaveric brain implanted with an isolated DBS lead. RF heating remained below 2°C, similar to a fever, with the 95% confidence interval between 0.38°C-0.52°C. Magnetic forces were well below forces imposed by gravity, and thus not a source of concern. No device malfunctioning was observed due to interference from MRI fields. Electrode artifact was most noticeable on MPRAGE and T2*GRE sequences, while it was minimized on T2-TSE images. Our work provides the safety assessment of ultra-high field MRI at 7 T in patients with DBS implants. Our results suggest that 7 T MRI may be performed safely in patients with DBS implants for specific implant models and MRI hardware.

## Introduction

Deep brain stimulation (DBS) therapy is among the most important advances in clinical neuroscience over the past two decades. DBS involves an initial neurosurgical procedure to implant electrodes into specific targets within the brain, that then deliver constant or intermittent electrical pulses via an implanted pulse generator (IPG) to modulate aberrant neural behavior. DBS is currently the gold standard treatment for drug-resistant Parkinson’s disease and essential tremor and has received humanitarian device exemption for treatment of dystonia and obsessive-compulsive disorder. In addition, there are numerous clinical trials currently underway or recently completed to evaluate the efficacy of DBS in treating other disorders, most notably chronic pain, epilepsy, major depression, and Alzheimer’s disease.

It is estimated that 70% of patients with DBS implants will need magnetic resonance imaging (MRI) within 10 years of their implantation [1]. Despite tremendous potential of MRI and fMRI to guide DBS therapy, safety concerns have limited post-operative accessibility of MRI to patients with DBS devices, mainly due to fatal injuries resulting from radiofrequency (RF) heating of DBS leads during the transmit phase of MRI [2]. For this reason, strict conditions have been put in place to perform MRI in DBS patients, namely, majority of DBS systems are approved for horizontal 1.5 T scanners using pulse sequences with B_1_^+^RMS<2μT [3–5].

To date, studies that have assessed the safety and feasibility of postoperative MRI for DBS imaging have focused on 1.5 T and 3 T scanners due to their clinical prevalence [2, 6–10]. Ultra-high field MRI at 7 T provides promise to push the boundaries of DBS target visualization [11], yet no study has assessed safety and image quality of 7 T MRI in patients with DBS devices. Here we report results of RF heating assessment, magnetic force measurement, and image artifact of a commercial DBS device implanted in an anthropomorphic phantom undergoing MRI at 7 T. RF heating measurements were performed with 43 unique device configurations based on realistic lead trajectories extracted from postoperative CT images of patients. A multi-material anthropomorphic phantom was constructed and used to maximize the resemblance of RF exposure experiments to what happens *in vivo*. Magnetic force measurements were performed according to ASTM F2052-15 [12]. Finally, we assessed the metal artifact around implanted leads in a cadaveric brain using imaging sequences optimized for DBS target visualization.

Our results provide relevant data to assess RF safety of MRI in patients with DBS devices.

## Materials and methods

### Magnetic resonance imaging equipment and imaging protocols

**Fig 1:**
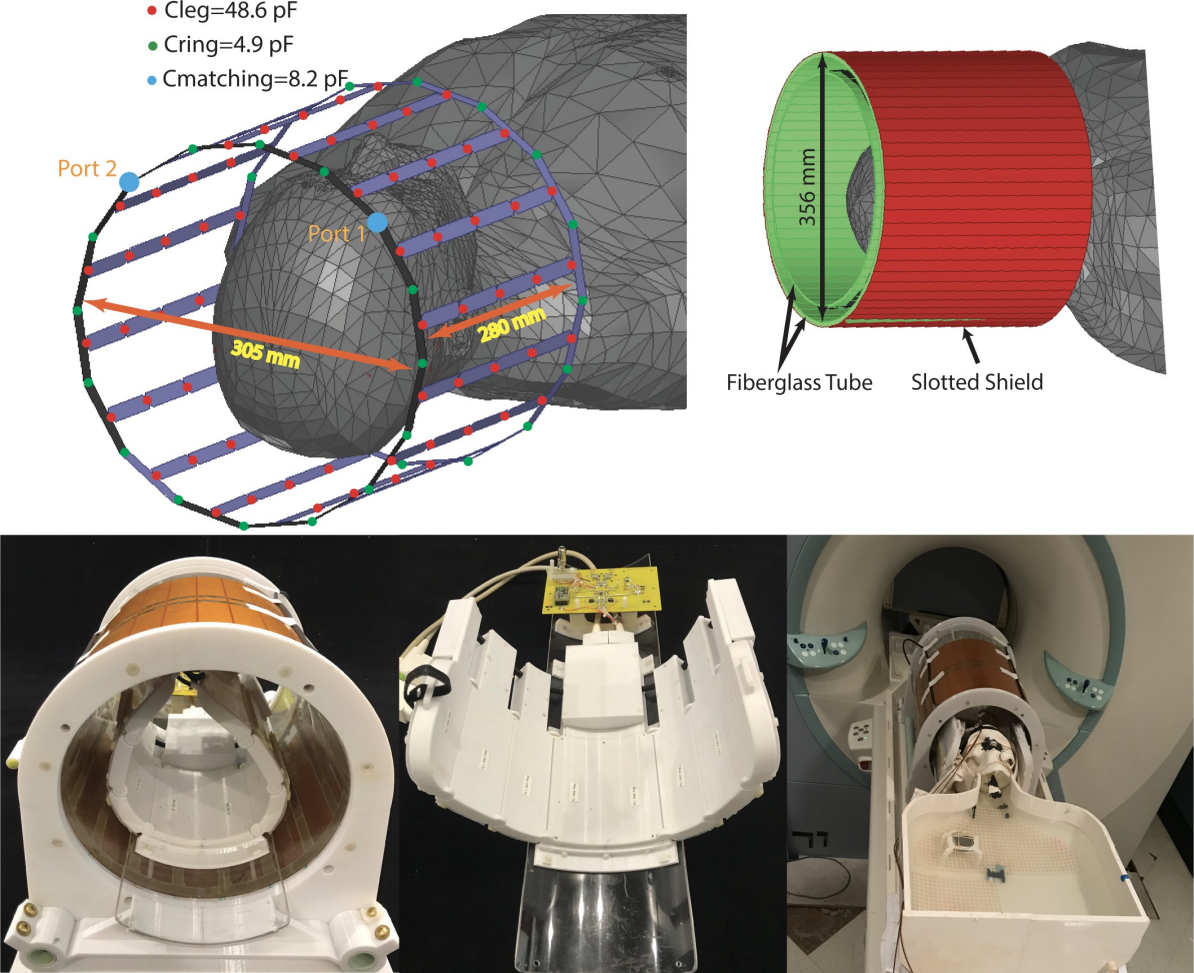
RF coil and phantom configurations.

Experiments were performed on a 7 T scanner (MAGNETOM; Siemens Healthineers, Erlangen, Germany) using a home-made local transmit/receive head coil. The coil used in experiments was an 8-channel wrap-around receive array, nested inside a shielded detunable quadrature birdcage volume coil (Fig 1). The volume coil slid (in the bore direction) to increase accessibility for the patient/phantom. The receive array helmet was sized to accommodate a majority of adult heads and was large enough to fit the phantom. It comprised of 8 overlapped rectangular elements (6.5 cm x 13 cm), built on 8 panels that were arranged in a u-shape around the head. A 3D-printed hinge mechanism used between neighboring panels allowed the array to be wrapped closely around phantom’s head to provide a high filling factor and maximal SNR. Flexible copper braid was used over the panel joints to allow the hinges to move without breaking each coil’s conducting path. Each loop was made of 16 AWG wire with seven or eight evenly-spaced tuning capacitors. All elements were tuned to 297.2 MHz and matched when loaded to an impedance of 75 Ω to minimize the noise figure of the Siemens 7T preamplifiers.

The transmit coil was a detunable 16-rung band-pass birdcage with a rung length of 28 cm and diameter of 30.5 cm which was nested inside a cylindrical slotted shield. Birdcage conductors were routed out of 7.8 mm circuit board and then bent and fastened to the inner surface of a fiberglass tube. The birdcage coil was tuned to 297.2 MHz using 16x4.9 pF capacitors distributed around each end ring and 5x48.6 pF capacitors distributed along each leg. Series capacitors of 8.2 pF were used to match the coil a loaded impedance of 50 Ω. Drive ports were located at the top front side of the coil (see Fig 1).

RF heating measurements were performed during 7 minutes of imaging with a T_2_W HASTE sequence (32 averages), where TE, TR and echo spacing parameters were adjusted to reach the maximum SAR allowed by the scanner. For B_1_^+^ estimation, we aquired flip angle maps in the phantom using Siemens’s tfl_WIP543_B1map pulse sequence with TE = 2.14 ms, TR = 2000 ms and nominal flip angle of 90°. The flip angle maps were then converted to maps of B_1_^+^/Volt which was the B_1_^+^ produced by the coil per unite of voltage exication. These maps were then used to estimate the B_1_^+^RMS during the HASTE sequence. HASTE sequence parameters for RF heating measurements are summarized in Table 1.

**Table 1:** Sequence parameters for HASTE sequence used for RF heating measurements.

T2W-HASTE	
TE (msec)	99
TR (msec)	2000
FOV (mm)	200
FA	180
Echo Spacing (msec)	4.96
Turbo Factor	319
TA	7:04
No of Slices	6
Slice thickness (mm)	2
Resolution (mm)	0.6x0.6x2.0
Averages	32
B_1_^+^RMS (*μT*)	2.88
Most Critical Safety Aspect	Local Head SAR 10 W/kg

TE = Echo time, TR = Repetition time, FOV = Field of view, FA = Flip Angle, TA = Acquisition time.

Electrode artifact was assessed on MPRAGE, T_2_W TSE and T_2_*W GRE images with parameters that were reported to optimize visualization of subthalamic nucleus in patients [13, 14]. Details of sequence parameters for image artifact assessment are reported in Table 2.

**Table 2:** Sequence parameters for image artifact study.

Parameters	Sequence		
	MPRAGE	T_2_W- TSE	T_2_*W-GRE
TE (msec)	1.74, 3.68	58	17.8
TR (msec)	2530	8000	501
FOV (mm)	165	163	176
FA	7	131	40
TA	8:53	9:28	6.27
No of Slices	224	80	17
Slice thickness (mm)	0.8	1	1
Resolution (mm)	0.8x0.8x0.8	0.4x0.4x1.0	0.5x0.5x1.0
Averages	2	2	2

TE = Echo time, TR = Repetition time, FOV = Field of view, FA = Flip Angle, TA = Acquisition time.

### Anthropomorphic phantom and DBS device configurations

It is well established that RF heating of elongated conductive implants (such as leads) is highly dependent on the trajectory of the implant and distribution of MRI electric fields in the sample and around the implant [15–22]. To have a more realistic field distribution around the implanted lead, we used a multi-material anthropomorphic phantom consisting of a 3D-printed body-shaped container and a refillable skull structure, with the design based on CT images of a patient with a DBS device. Use of patient data for the purpose of MRI RF heating assessment and publication of de-identified images was approved by Northwestern University’s Institutional Review Board (STU00206859). Consent was waived as the data was collected retrospectively and analyzed anonymously.

Details of phantom design and construction are described elsewhere [23]. In brief, the skull was filled with a tissue mimicking gel, prepared by mixing 32 g/L of edible agar (Landor Trading Company, gel strength 900 g/cm2) with saline solution (2.25 gNaCL/L) with electric conductivity of *σ* = 0.43 *S*/*m*, similar to values reported for brain tissue [24]. Once cooled down to room temperature, the mixture formed a semi-solid gel which kept the DBS lead in place, and had the thermal conductivity of ~0.56 J/K-S [25] similar to that of grey matter [26].

**Fig 2:**
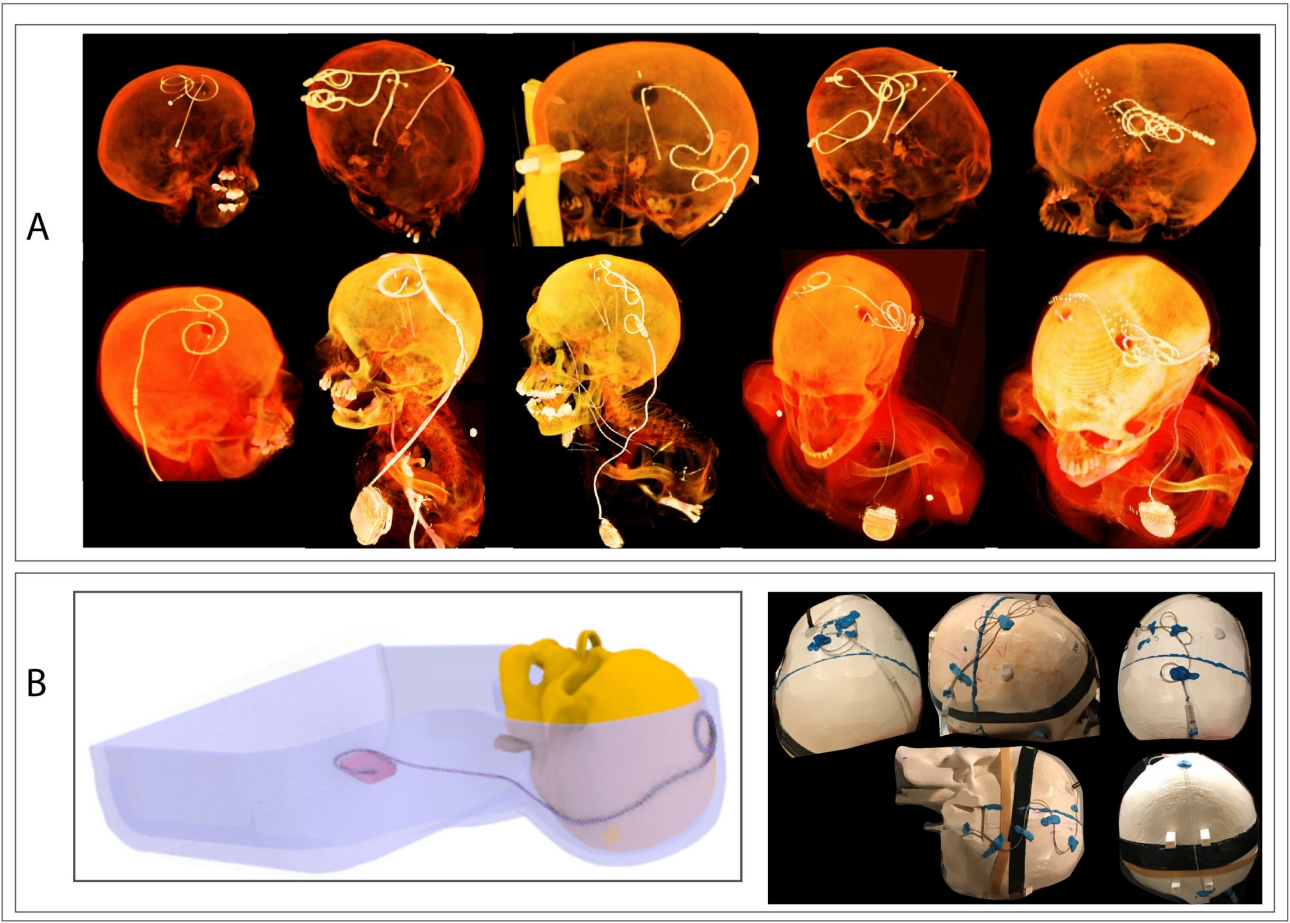
DBS trajectory configurations. (A) 3D-rendered postoperative CT images of DBS patients showing typical trajectories of extra-cranial leads and extensions. (B) Schematic of the phantom implanted with a full DBS device as well as examples of some trajectories used in RF heating experiments.

DBS surgery is usually performed at two stages: first, electrodes are implanted in the target nuclei with the extracranial portion of the lead routed under the scalp, and the patient is sent home to recover. In a second surgery leads are connected to extensions, which are then routed subcutaneously down the neck toward the pectoral region and connected to the IPG. MRI in patients with lead-only systems is useful for target verification, but a majority of MRI exams, including those that study functional effect of stimulation, are performed in patients with fully implanted DBS systems. We replicated 28 configurations for lead-only systems (11 configurations with Medtronic lead 3387, and 17 configurations with Medtronic lead 3389) and 15 configurations for a fully implanted DBS system (Medtronic lead 3389, extension 3708660, IPG Activa SC-37603) for RF heating experiments. Fig 2 shows typical DBS lead trajectories from post-operative CT images of patients as well as some that were replicated in the phantom.

### RF heating measurements

**Fig 3:**
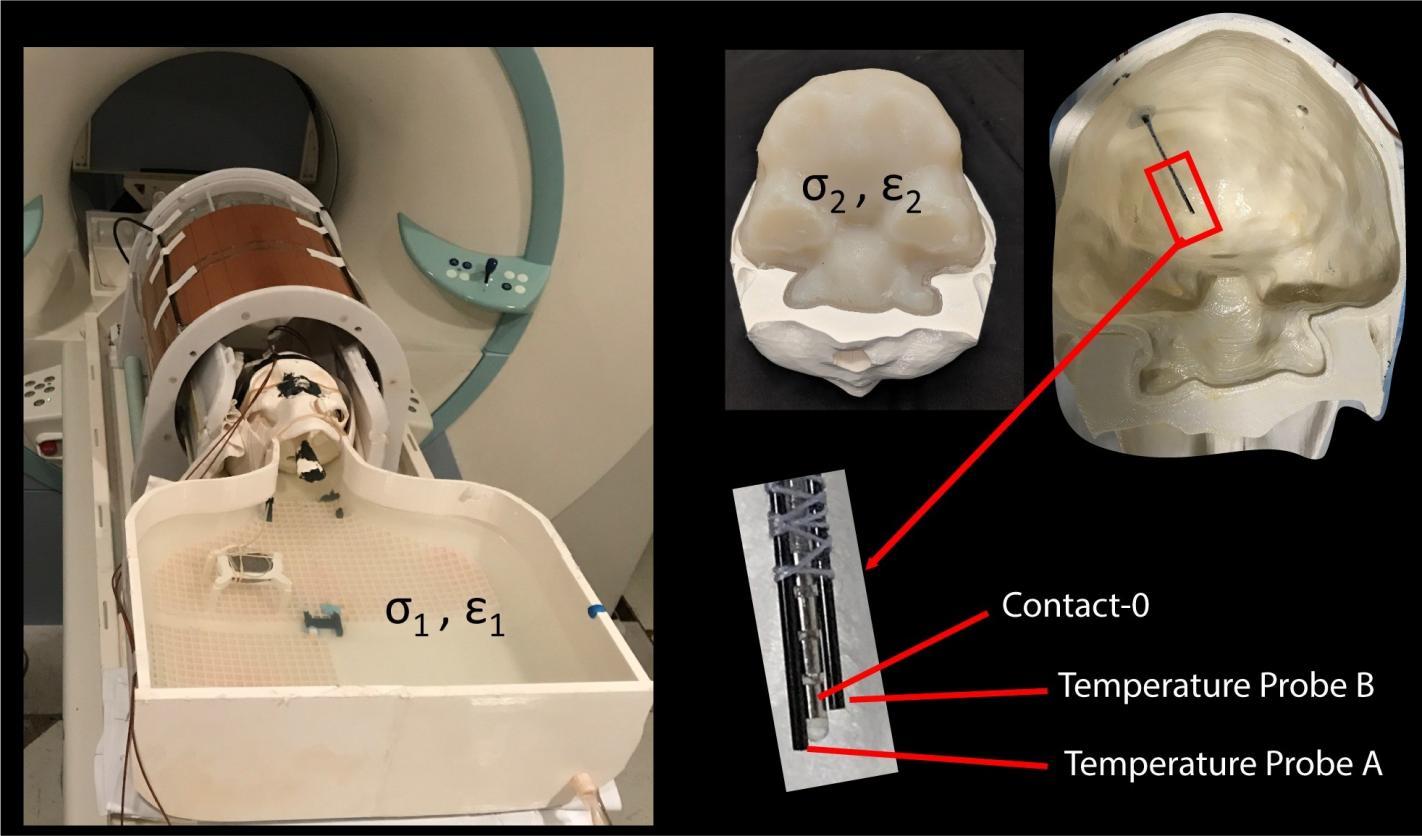
Experimental setup for RF heating measurements. Experimental setup with saline-filled anthropomorphic phantom (*ε*_1_ = 78, *σ*_1_ = 0.51 *S*/*m*) and gel-filled skull (*ε*_2_ = 75, *σ*_2_ = 0.43 *S*/*m*). Positioning of temperature probes at the tip of lead model 3389 is also shown.

Two MR-compatible fluoroptic temperature probes (OSENSA, BC, Canada, resolution 0.01°C) were secured close to electrode contact 0 of Medtronic DBS leads (Medtronic model 3387 or 3389) as shown in Fig 3. One probe was positioned such that it was in touch with the electrode contact itself, and the other was secured one millimeter further distally such that its tip was in contact with the gel. This allowed us to assess the spatial profile of RF heating around the DBS lead’s tip. The ensemble of lead-probe system was inserted into the gel-filled skull through a 5 mm hole following entry point, angle, and penetration depth analogous to the clinical approach for targeting the left subthalamic nucleus. The skull structure containing the lead-probe system was inserted into the phantom’s body container which was then filled with the saline solution (conductivity of *σ* = 0.51 *S*/*m* representing average tissue). In cases with a complete DBS system, the lead was connected to an extension (Medtronic model 3708660) and an implanted pulse generator (IPG) (Medtronic Activa SC-37603), with the IPG positioned either at the right pectoral region to create contralateral configuration or at the left pectoral region to create an ipsilateral configuration. The former (contralateral configuration) is shown to generally generate higher RF heating [27].

### Magnetic force measurements

**Fig 4:**
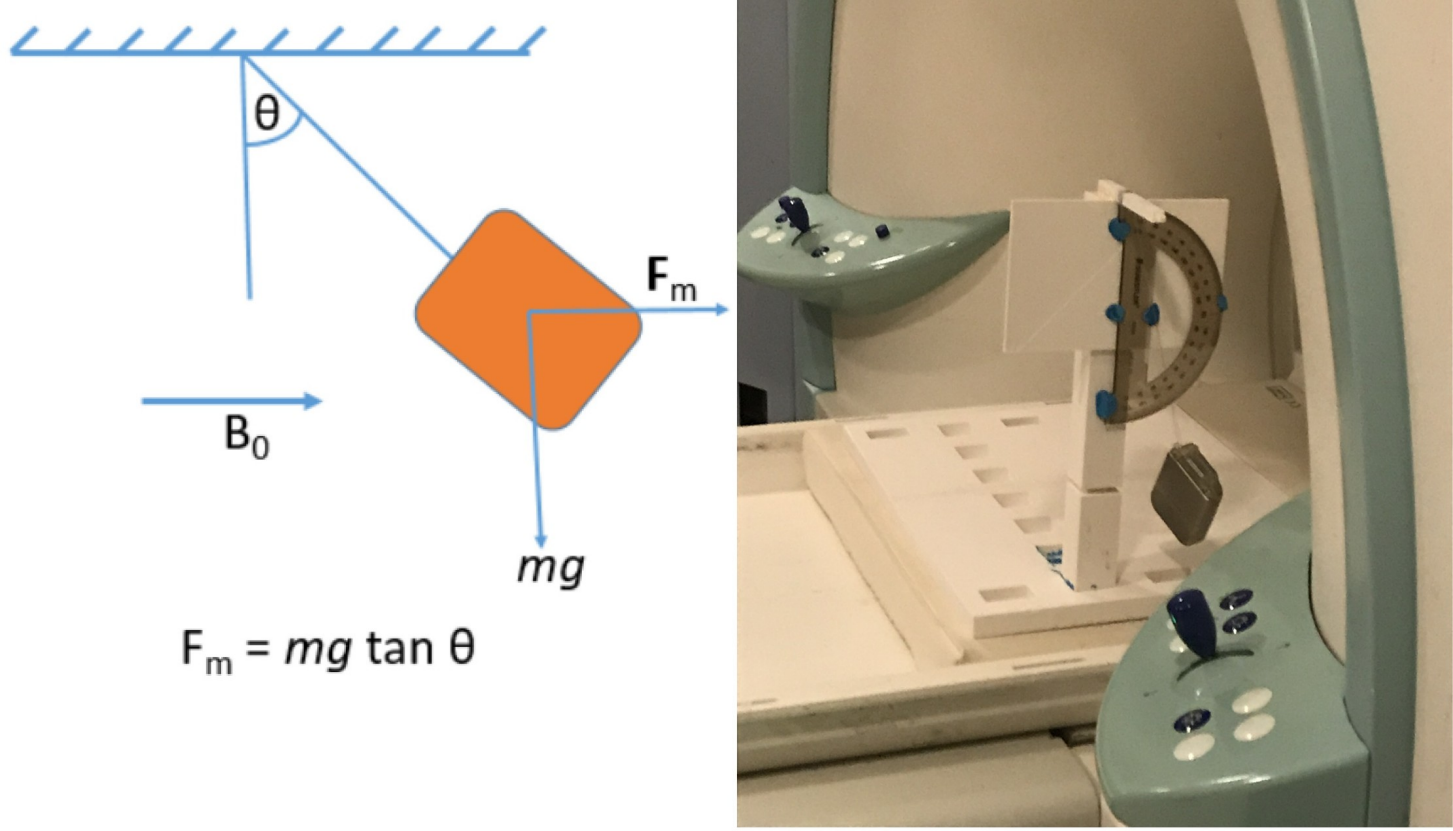
Force measurement setup.

Magnetically induced displacement force on the pulse generator produced by the spatial gradient of the static magnetic field was measured according to ASTM F2052 test method [12]. A home-made test fixture was designed and fabricated consisting of a nonmagnetic 3D-printed sturdy structure capable of holding the IPG in proper position without deflection (see Fig 4). The IPGs (Medtronic Activa PC-37601 and Activa SC-37603) were suspended (one at a time) by a string and the angular deflection of the string from the vertical was measured at a location near the entrance of the scanner bore where the spatial gradient of Δ***B***_**0**_ = *dB*_0_/*dz* was maximum as well as several other locations along the bore. If the device deflects less than 45°, then the magnetically induced deflection force is less than the force on the device due to gravity (its weight). For this condition, it can be assumed that any risk imposed by the application of the magnetically induced force is no greater than any risk imposed by normal daily activity in the Earth’s gravitational field.

### Image artifact

**Fig 5:**
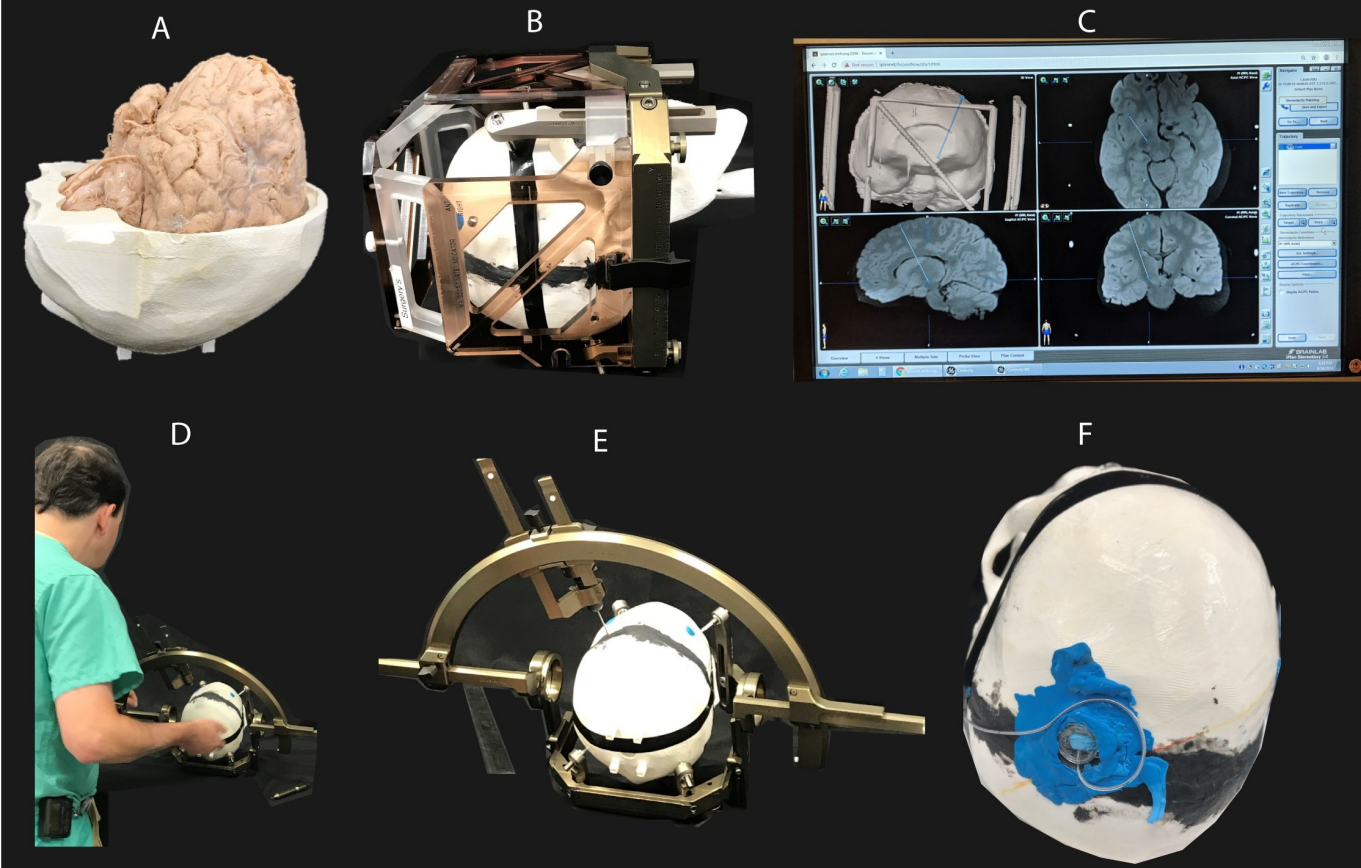
Steps of implanting DBS lead into a cadaver brain. (A) A formalin-fixed cadaveric brain was contained inside a 3D printed skull structure which was then filled with formalin solution. (B) The skull was attached to a Lakesell model G base ring (Elekta, Stockholm, Sweden) using titanium fixation pins, placed inside MRI fiducial box, and scanned at 1.5T for localizing subthalamic nucleus. (C) Images from MRI scan were transferred to BrainLAB iPlan server for determining coordinates of subthalamic nucleus as well as the entry point of the DBS lead on the skull. (D-F) A StimLoc ring was attached to the skull for the electrode implantation. After implanting the DBS lead, the StimLock ring was removed and a burr hole cover was placed to fixate the lead at the entry point.

To assess the image artifact, the gel-filled skull was replaced with a similar 3D-printed skull structure which contained a formalin-fixed cadaveric brain which was donated to Northwestern Medical School for educational and research purposes. None of the transplant donors were from a vulnerable population and all donors or next of kin provided written informed consent that was freely given. An isolated Medtronic DBS lead (model 3387) was implanted into the subthalamic nucleus of the cadaver brain as described in previous work [28]. Steps of preparing and implanting the DBS into the cadaver brain are given in Fig 5. The brain-containing skull was then placed inside the saline-filled torso of the phantom and scanned using MPRAGE, T_2_W-TSE, and T_2_*W-GRE sequences with details given in Table 2. Images were imported into 3D slicer (Slicer 4.10, www.slicer.org) and the width of the artifact around the DBS contacts was measured manually.

### Assessment of DBS system integrity

Before starting the experiments, we verified the integrity of the implanted DBS system by measuring the inter-electrode as well as electrode‐IPG impedances (for fully implanted systems) as recommended by the manufacturer. The IPG was set to “stimulation off” during experiments. Impedance measurements were also repeated after MRI experiments to ensure RF exposure did not impair the device function.

## Results

### RF heating

Table 3 gives the temperature rise at tips of leads 3389 and 3387 configured along different trajectories, measured by probe A at the end of a 7-minute scan with the T_2_W HASTE sequence. The maximum difference in recorded temperatures by probes A and B was less than 0.06°C. The mean±std temperature rise Δ*T* was 0.58±0.23°C for the lead-only system with lead 3387, 0.57±0.30°C for the lead-only system with lead 3389, and 0.52±0.32°C for the full system (lead 3389+extension 3708660+IPG Activa PC-37601). A single factor ANOVA showed no significant difference in the Δ*T* between groups (P-value = 0.84).

**Table 3:** Temperature rise for lead-only and full DBS configurations.

Lead only-3389		Lead only-3387		Full system	
config. #	Δ*T* [°C]	config. #	Δ*T* [°C]	config. #	Δ*T* [°C]
1	1.06	1	0.68	1	1.01
2	0.54	2	1.08	2	0.22
3	0.8	3	0.6	3	0.37
4	0.42	4	0.59	4	0.77
5	0.22	5	0.46	5	0.49
6	0.52	6	0.34	6	1.07
7	0.35	7	0.89	7	0.63
8	0.71	8	0.34	8	0.45
9	0.51	9	0.58	9	0.24
10	0.46	10	0.38	10	0.22
11	1.36	11	0.47	11	0.31
12	0.27	-	-	12	0.19
13	0.3	-	-	13	0.17
14	0.84	-	-	14	0.86
15	0.45	-	-	15	0.84
16	0.56	-	-	-	-
17	0.35	-	-	-	-

**Fig 6:**
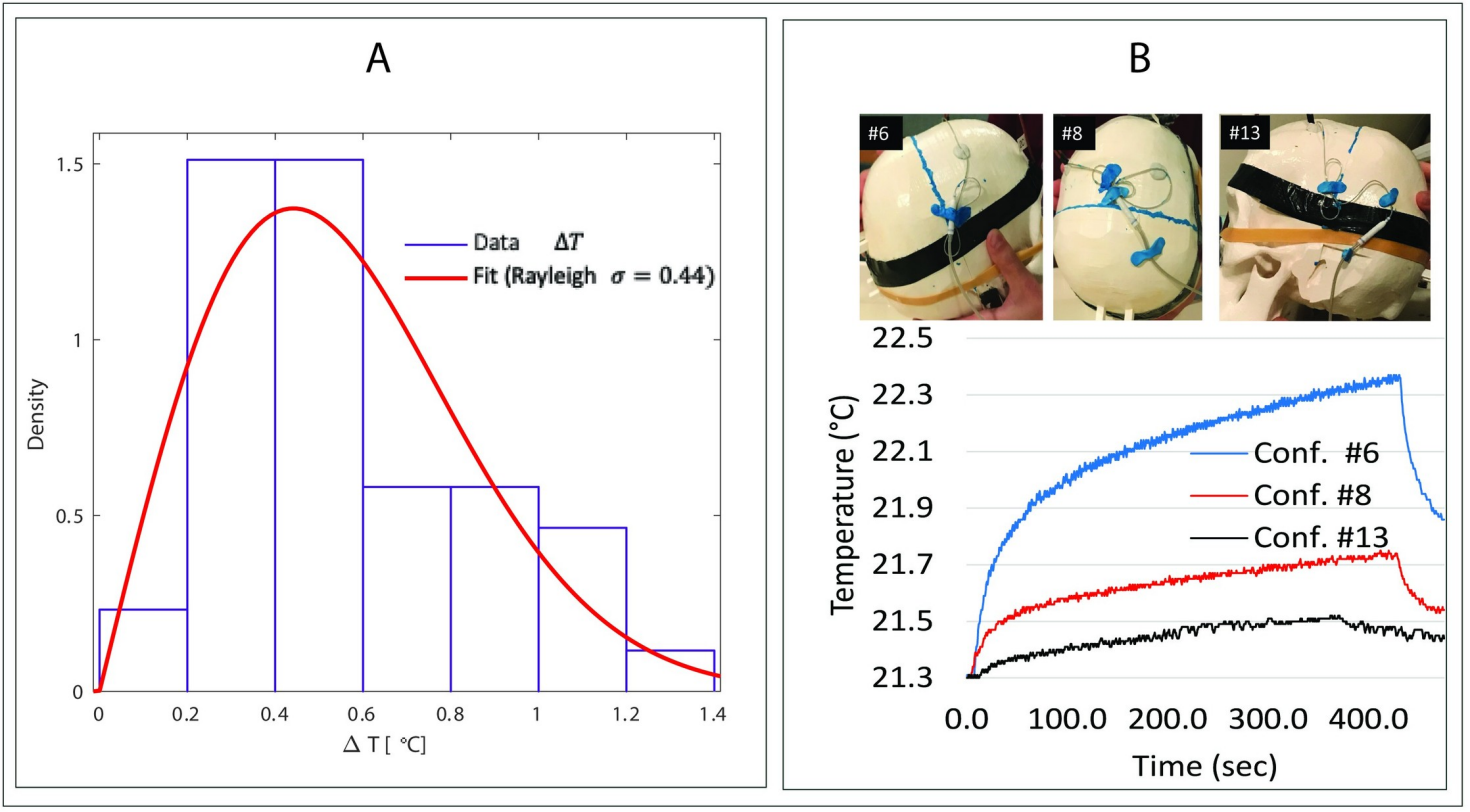
RF heating results. (A) Distribution of pooled data (n = 43) fitted to a Rayleigh probability distribution MATLAB. (B) Temporal profile of temperature rise for three configurations (Conf #6, #8 and #13 as shown in the attached pictures) for full DBS system, representing maximum, intermediate and the minimum heating scenarios.

Fig 6A shows distribution of the pooled data (Δ*T* from all three groups, n = 43) fitted to a Rayleigh probability distribution (*σ* = 0.44, standard error = 0.03) using MATLAB R2019a *Distribution Fitter* app. Under this distribution, the 95% confidence interval of Δ*T* is estimated to fall between 0.38°C-0.52°C. Fig 6B shows lead configurations that generated the maximum (Δ*T* = 1.4°C), minimum (Δ*T* = 0.2°C), and average (Δ*T* = 0.5°C) temperature rise for the fully implanted DBS system, along with their temporal heating profile.

### Induced magnetic force and system integrity

The maximum deflection angle was observed when the IPG was hung close to the scanner wall at the entrance of the bore and was measured to be 25° for the IPG model Activa PC (Model 37601) and 36° for the model Activa SC (Model 37603). This means that the magnetic forces experienced during the scan do not pose a risk greater than the exposure of the device to Earth’s gravitational forces. Measured impedance values before and after RF heating experiments were within the manufacturer’s recommended range, namely <4000 Ω for inter-electrode impedances and <2000 Ω for electrode-IPG impedances and also with no short circuits.

### Image artifact

**Fig 7:**
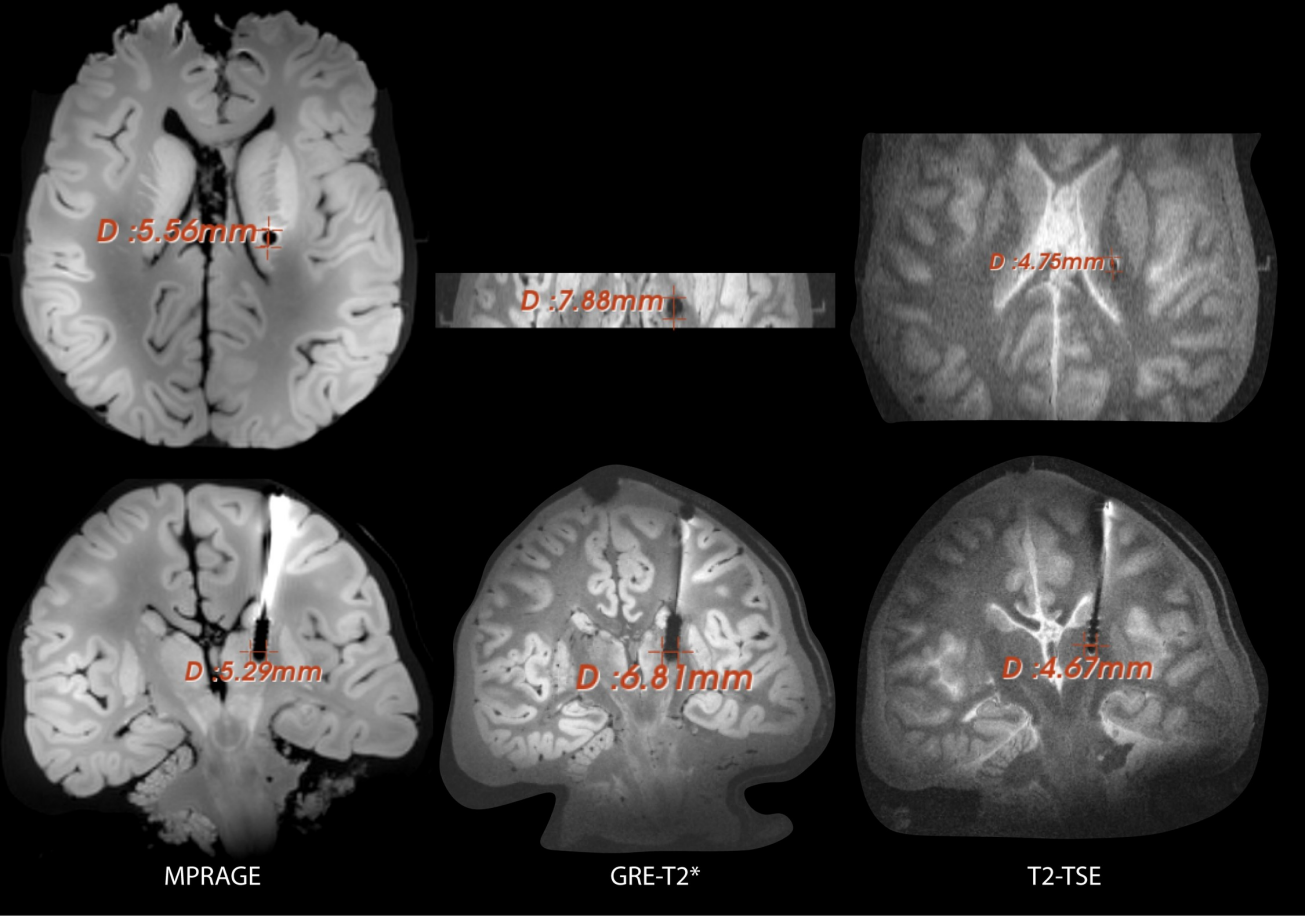
Image artifact measurements. Image artifact around the DBS lead implanted into the cadaveric brain. Each column represents the artifact in transverse and coronal planes for sequences detailed in Table 2. The artifact size (width) around electrode contacts is reported for each case. Scans were performed with a lead-only system, with the extra-cranial portion of the lead looped around the bur hole.

The image artifacts around the DBS lead produced during MRI imaging with different routine clinical sequences (Table 2) are shown in the Fig 7. The artifact diameter around the electrode contacts of the DBS lead were measured on transverse as well as coronal planes as depicted in the Fig 7. The average size of the artifact (averaged between transverse and coronal slice for each sequence) around the DBS contacts was observed to be 4.7 mm, 5.4 mm and 7.3 mm for the sequences T2-TSE, MPRAGE and T2*-GRE respectively.

## Discussion

The rising prevalence of chronic diseases coupled with the rapidly aging population worldwide has made medical implants more ubiquitous than ever [29, 30]. More than 12 million Americans currently carry a form of orthopedic, cardiovascular, or neuro-modulation device and the number grows by 80,000 annually. It is estimated that 50%-75% of patients with conductive implants will need to undergo MRI during their lifetime [31], with many patients requiring repeated examinations [32].

Advances in device engineering have led to a new generation of electronic implants that are largely immune to MRI-generated static and gradient fields. Tissue heating from radiofrequency (RF) excitation fields, however, remains a major issue. This *“antenna effect”* [33–35] happens when the electric field of the MRI transmitter couples with implanted leads, causing the specific absorption rate (SAR) of the RF energy to significantly amplify at the implant’s tip [36]. Given the potential for fatal hazards, the conditions under which patients with conductive implants are indicated for MRI are restrictive. In patients with DBS devices for example, MRI is allowed only at 1.5T field strength for majority of devices, using pulse sequences with a SAR of 0.1 W/kg (30 times below FDA limit for scanning in absence of implants) or B_1_^+^RMS<2μT, and the current state-of-the-art neuroimaging techniques at 3T and above are contraindicated [3–5]. There is strong incentive, however, toward use of high and ultra-high field MRI to inform DBS therapy:1.) 1.5T MRI systematically underestimates the anterior and lateral boundaries of the subthalamic nucleus (STN) and globus pallidus (common DBS targets) compared to fast gray matter T1 inversion recovery acquisitions at higher fields [37], and 2.) high-field MRI confers a much better contrast-to-noise ratio which makes it easier to delineate small abutting structures [38]. Given that DBS targets such as the STN, are bordered by several small structures (e.g, the ansa lenticularis, zona incerta, and substanitia nigra), this capacity becomes crucial. Finally, high-field MRI is more sensitive to susceptibility artifacts which acts as an advantage when visualizing the iron-rich STN [39, 40] and helps delineate pallidofugal and striatonigral fiber tracts, further aiding electrode positioning [41].

The past few years have witnessed a spike in engineering efforts to realize *implant-friendly* MRI for patients with DBS implants. Pioneering work has been done to advance MRI field-shaping methods,—techniques that manipulate the electric field of MRI transmit coil to eliminate its interaction with individual patient’s implants [6, 8, 42–49]. Another alternative approach is surgical device management, where trajectories of implanted leads are surgically modified (based on computer simulations) such that their coupling with MRI electric fields is minimized [21, 50]. Finally, there has been a spate of patents and papers proposing novel implant structures [51–55] and materials [56, 57] to reduce induced RF currents, and by proxy, RF heating. Despite the promising potential of these innovations to make state-of-the-art MRI accessible to patients with DBS implants, their clinical translation has been very slow. As a result, researchers have resorted to application of MRI beyond the currently approved labeling of the device based on RF heating assessment through phantom experiments or numerical simulations. These studies, however, have focused on 1.2T, 1.5T and 3T scanners due to their clinical prevalence [10, 58–62]. This work presents the first study of MRI safety in patients with DBS implants at 7T, and as such, provides a frame of reference to compare risks and benefits of ultra-high field MRI in this patient population.

The technical specification ISO-TS 10974 provides insights into different mechanisms that could potentially cause hazard during MRI in patients with active electronic implants [63]. Specifically, most prominent sources of hazard known today are electrode heating, unintended stimulation, device heating, device malfunction and device vibration, as well as dislodgment due to magnetic force and torque. In terms of electrode heating, our results show that at 7T, the maximum heating for a wide range of clinically relevant as well as worst-case scenarios remains well below 2°C, similar to a fever.

Other sources of hazard include device vibration and/or malfunction, device dislodgement due to magnetic forces, and unintended stimulation. Unintended tissue stimulation is a source of concern where leads are in touch with cardiac tissue, such as in cardiovascular implanted electronic devices, as stimulation could cause arrythmia. In DBS patients, such effect has not been observed [58] and may not be a potential concern. Device vibration could happen due to MRI gradient fields and is only relevant for implants with large conductive plates and not a source hazard for implanted leads [63]. Also, in our experiments, we did not notice any vibration in the IPG during the scans. With regard to magnetic force, our measurements showed that such forces were well below the limit that could potentially pose any risk. Finally, for the specific device model that was tested, system integrity verification ruled out the possibility of device malfunction due to damages caused by MRI fields. We would like to note, however, that results of this study should not be interpreted as proof for safety of scanning patients with DBS devices at 7T in a similar way that MR-conditional labeling of such devices do. Specifically, more tests are required to assure all aspects of patient and device safety are evaluated as laid out in ISO-TS 10974. This result of this work should be only interpreted as a preliminary assessment that would incentivize performing more thorough tests that lead to labeling of DBS devices at 7T.

In terms of image quality, 7T MRI is shown to allow direct and clear visualization of the small and deep cortical structures such as the STN—a main target in DBS for treating Parkinson’s disease [11, 13]. However, as the high field MRI is prone to increased susceptibility artifacts, the image artifacts around the DBS contacts could be a drawback of using 7T MRI for target verification. For the particular model of DBS lead used in this study, we observed that the artifact size (4-6mm) was comparable to the size of the STN [64]. Which means, for a properly positioned DBS lead, the STN or similar sized targets might be fully or mostly masked by the artifact. However, due to clear direct visualization of the neighboring structures, such as substantia nigra at 7 T, it may be still possible to verify the position of the DBS lead relative to the target nucleus. Specifically, Fig 7 shows a substantially smaller artifact for a spin echo sequence (T2-TSE in Table 2) compared to gradient echo or MPRAGE as recommended in the earlier study [65]. Also, as the size of the artifact may depend upon the lead geometry, the results may differ among lead models.

## Conclusion

This work provides the first safety assessment of ultra-high field MRI at 7T in patients with DBS implants. Our results suggest that 7T MRI may be performed safely in patients with DBS implants for specific implant models and MRI hardware. However, these results should not be generalized to other models of DBS implants that have not been tested here, or to other 7T MRI hardware.
